# Effects of contextual interference and differential learning on performance and mental representations in a golf putting task

**DOI:** 10.1002/ejsc.12079

**Published:** 2024-02-13

**Authors:** S. Hourieh Mousavi, Alireza Saberi Kakhki, Davoud Fazeli, Ludwig Vogel, Fabian Horst, Wolfgang I. Schöllhorn

**Affiliations:** ^1^ Department of Motor Behavior Ferdowsi University of Mashhad (FUM) Mashhad Iran; ^2^ Department of Sport Sciences Faculty of Education and Psychology Shiraz University Shiraz Iran; ^3^ “Neurocognition and Action ‐ Biomechanics” Research Group Bielefeld University Bielefeld Germany; ^4^ Center for Cognitive Interaction Technology (CITEC) Bielefeld University Bielefeld Germany; ^5^ Department of Training and Movement Sciences Institute of Sport Science Johannes Gutenberg University of Mainz Mainz Germany

**Keywords:** motor learning, motor noise, perceptual‐cognitive perspective, practice schedule, stochastic resonance

## Abstract

It is widely accepted that mental representations can have an important influence on motor performance. Although differences in mental representations of motor tasks have been reported between novices and experts, little is known about their development as a function of motor learning approaches. The aim of this study was to compare the effects of contextual interference (CI) and differential learning (DL) on the performance and mental representations in a golf putting task. A total of 40 participants were randomly assigned into four groups: blocked contextual interference (BCI), random contextual interference (RCI), DL, and control. First, the participant's initial mental representation level was tested by means of the structural dimensional analysis of mental representation. Then, the participant's initial performance level was tested by 12 golf‐putting trials from 2.44 m. During the acquisition phase, participants practiced golf putting according to their grouping for three consecutive days with 10 blocks of 12 trials per day. No intervention was applied for the control group. The retention‐tests were performed 72 h after the last acquisition day. In addition, a transfer test to a novel distance outside the acquired range (4 m) was performed immediately after the retention‐test. The results of the putting performance in the retention test showed that RCI and DL performed better compared to BCI and the control group (all *p* < 0.05). In the transfer test, BCI and RCI outperformed the control group (all *p* < 0.05), but both were further outperformed by the DL group (all *p* < 0.05). Moreover, the DL group showed a more structured mental representation than the other groups during the retention test. These results indicated that DL used a different underlying mechanism that resulted in different levels of performance during transfer and a more structured mental representation compared with CI.

## Highlights

- There was no significant difference between random contextual interference (RCI) and differential learning (DL) groups during retention.
- Both RCI and DL groups outperformed the blocked contextual interference (BCI) group in the retention test.
- The DL group performed more accurately than the BCI and RCI groups during the transfer test.
- The DL group had a more structured mental representation than the BCI and RCI groups.

## 1 INTRODUCTION

In the psychology of motor learning, a distinction between three perspectives can be made: central (i.e., cognitive), peripheral (i.e., ecological), and perceptual‐cognitive perspectives (Schack et al., 2021). Cognitive or central‐oriented perspectives are typically modeled by means of internal representations for example, motor program (Keele, 1968) and schema (Schmidt, 1975). However, peripheral‐oriented perspectives consider motor learning as an emergent property of interactions between learners and the environment (McMorris, 2014). In contrast, the perceptual‐cognitive perspective believes that motor actions are guided via representations that contain information about the perceptual effects of motor actions (Schack, 2004).

The cognitive and peripheral perspectives propose different practical consequences for motor learning. Although both perspectives suggest benefits for variable over repetitive motor learning, the way variability is theoretically derived and implemented in motor learning approaches differs. Cognitive perspectives introduce variability in blocked or random and serial practice (contextual interference [CI]) (Shea et al., 1979) schedules. While in the blocked schedule (variability of practice [VP]) the precisely prescribed exercises are only changed after several repetitions to stabilize the pattern of a single movement (Moxley, 1979; Schmidt, 1975), in the latter there is a random or cyclical change between a certain number of defined exercises, all of which must be stabilized in parallel. The blocked schedule is also referred to as low CI and the other two as high CI. Thereby, the variation within the same generalized motor program (GMP) is to be distinguished from varying between different GMPs. The larger effects are associated with the latter (Wulf et al., 2002), especially in movements that have a small number of degrees of freedom (sDGF). Golf putting from different distances would correspond to the first, whereas switching between drive, chipping, and putting would correspond to the latter (Shea et al., 1979). However, studies on movements with a large number of degrees of freedom (lDGF) varying within the same GMP seem to be more advantageous (Apidogo et al., 2022).

The CI‐effect (Shea et al., 1979) is linked to two phenomena: a performance decline after acquisition and a subsequent improvement during retention. The initial decline is attributed to working memory (WM) overload due to increased exercises to be remembered. Two hypotheses, elaboration and distinctiveness (Shea et al., 1979) and forgetting‐reconstruction (Shea et al., 1983), try to explain the second part. Elaboration emphasizes random practice for task comparison, enhancing a structured mental representation. Forgetting‐reconstruction suggests forgetting the previous action plan during random practice, leading to a more structured mental representation compared to blocked practice. Both align with the cognitive perspective, focusing on forming central motor programs through a large number of “correct” repetitions. In the CI model, errors are traditionally viewed as detrimental to learning (Shea et al., 1983; Wulf et al., 1988). Target errors in aiming tasks may lead to frustration (Rendell et al., 2010) and to additional load for the limited WM (Broadbent et al., 2017). The latter findings tended to be confirmed in a study in which CI was combined with an errorless learning strategy in the form of golf putting shots from distances that were increasing (Ramezanzade et al., 2022). The term errorless is confusing here, as a certain degree of movement deviations from a short distance is considered errorless, whereas the same deviations of the movements from a greater distance are judged as errors.

Furthermore, the distinction between movement and target errors is particularly important for the processing of different feedback characteristic. This issue corresponds to the distinction between knowledge of result (KR) and knowledge of performance (KP) in feedback research and is also closely linked to their associated problems (Micallef et al., 2023; Newell, 1991; Oppici et al., 2021). If a learner receives feedback in the form of KR (e.g., hitting the hole or not) for movements with a larger number of degrees of freedom, the learner is faced with the problem of deciding between numerous possible solutions, which is typically associated with an additional burden on the WM and in the end corresponds to trial‐and‐error learning. In contrast, with KP (e.g., swinging too fast in the beginning but too slow at the end) the decision is simplified, as the learner receives direct information about the specific feature to be changed (Newell, 1991). Regardless of the type, providing feedback after positive attempts is ascribed a supportive role (Chiviacow et al., 2007; Saemi et al., 2012) and subsequently assigns errors the originally negative co‐notation in learning. Hereby, it is important to be aware that this right–wrong dichotomy encounters fundamental problems when we suggest something supposedly correct, due to the recent identification of individual movement patterns and the low probability of identical movement repetitions in movements with lDGF (Horst et al., 2016, 2017). Analogously systematic evidence for the generalization of the VP and CI model from movements with sDGF to movements with lDGF has not been succeeded yet (Ammar et al., 2023; Brady, 2004; Schöllhorn et al., 2022). Until the numerous contradictory findings are resolved by modifying the CI model, further boundary conditions of applicability need to be clarified (Schöllhorn, 2016). Recent studies comparing the CI model with the differential learning (DL) model (Apidogo et al., 2021, 2022) provide initial indications of modifications that mainly relate to a reinterpretation of the phenomenon of variance that is related to problems of the explanation models and observable during the repetition of movements and had been described similar with the spacing effect (Bjork et al., 1970), namely the training of neural nets and the constant change of livings beings by time (Schöllhorn et al., 2022).

In distinction to the CI model, originated in traditional cognitive psychology, the roots of the DL model lie in system dynamics, neurophysiology, and the field of AI (Schöllhorn, 2000). In contrast to cognition psychological learning models, the system dynamic theory views fluctuations in living systems as essential, attributing them a constructive character and considering their increase as a condition for guiding systems through phase transitions (Savelsbergh et al., 2010; Schöner et al., 1988). This noise, with varying color spectrums, triggers self‐organization by only supplying energy, allowing the system to find its own structure. Self‐organization or guided self‐organization, in this context, means local interactions are not explicitly guided by an external agent (Prokopenko et al., 2014). When movements are explicitly restricted or guided, as in the constraints‐led approach (Gray, 2020), it involves guided teaching. Unlike previous views on movement fluctuations, DL actively adds noise during repetitions, akin to training artificial neural nets, promoting resilience to external disturbances (Haykin, 1994; Schöllhorn et al., 2022). DL, broadly defined, encompasses chaotic, gradual, and repetitive learning with the latter minimizing differences in successive movements (Henz et al., 2018; Schöllhorn, 2016). Thereby, the origin of noise, internal or external, is of secondary interest (Schöllhorn et al., 2006). Noise‐like variations in practice broaden possible solutions, offering valuable experience for learners to navigate deviations and return to effective solutions. DL better equips learners for adaptation and exploration of individually optimized solutions, leading to increased acquisition and learning rates compared to repetitive learning (Schöllhorn et al., 2022; Wagner et al., 2008).

A recent study comparing random CI and chaotic DL in Volleyball novices found evidence supporting the superiority of DL in acquisition and learning (Apidogo et al., 2022). The DL group, despite practicing techniques with additional noise and never adhering to “correct” methods, outperformed the CI group significantly in both posttest and retention tests. The DL group's increased variety of exercises did not negatively impact the performance, contrary to expectations based on the CI model.

In a study (Schmidt et al., 2021) examining golf putting movement stabilization in novices over 4 weeks, no differences were observed between increasing contextual interference (CI) (Porter et al., 2010) and two variants of DL in posttests and two retention tests. The CI group varied putting distances weekly, while one DL group introduced wild variations in angles, angular velocities, accelerations, and distances throughout the intervention. Surprisingly, the interference observed in the CI group during acquisition, compared to the repetitive control group, was absent. Despite the DL theory recommending variations primarily in geometry for novices (Schöllhorn, 1999), the DL groups with wild and infinite variations showed comparable performance improvement to increasing CI. The stochastic resonance model in the DL model suggests a learning process involving the attunement of stochastic signals (Schöllhorn et al., 2004), adapting exercise selection noise to the athlete's noise for optimal learning (Schöllhorn, 1999, 2005). This aligns with the challenge point theory (Guadagnoli et al., 2004), translating optimal subjective information from cybernetic pedagogy (Ashby, 1956; Pask et al., 1972) for sports practitioners (Scott et al., 2007). Unlike the challenge point metaphor, the DL model considers individual prerequisites and situational conditions, offering quantitative investigation possibilities through physics‐based models such as stochastic resonance and noise.

Meanwhile, another perspective has been introduced to study motor learning and control by extending the cognitive with the perceptual domain, the perceptual‐cognitive perspective (Schack, 2004). This can be understood as an attempt to integrate the two previous perspectives. According to this view, motor learning results from a change, modification, and development of representation structure in long‐term memory, which contain information about the perceptual effects of actions (Meier et al., 2020; Schack et al., 2021). The structural dimensional analysis of mental representation (SDA‐M) was employed to assess mental representation structures of different motor actions (Fazeli et al., 2017; Lex et al., 2015; Schack et al., 2007). Generally, it was found that skilled performers have a more structured mental representation than novices. There are few studies addressing changes in mental representation due to different practice methods. For example, the changes in mental representation following random and blocked practice were addressed in golf putting task (Fazeli et al., 2017). Participants practiced a putting task for seven consecutive days. Results showed that random practice would result in more accurate performance and a more structured mental representation compared with blocked practice.

Although CI and DL have been evaluated separately, they have rarely been evaluated simultaneously in a single research paradigm. For example, (Henz et al., 2018) and (Serrien et al., 2020) are two studies that compared neuronal or performance effects of these two learning approaches directly. However, in these studies, only the short‐term effects have been studied, medium‐ or long‐term effects have not been considered. In the present study, medium‐term effects of both approaches will be compared. One study on CI learning has been conducted to investigate the structure of mental representation (Fazeli et al., 2017); however, in the context of DL, no study of possible effects on mental representations was found. Some studies showed differences in neural activity between CI and DL. For example, (Henz et al., 2016) and (Henz et al., 2018) showed that DL engaged more regions of the cortex than repetitive learning or CI. These findings could be considered as evidence for the role of unconscious cognition in the DL method. However, some operators of dynamic systems theory do deny a role of cognitive levels in movement and others try to redefine cognition from a non‐representational point of view (Schack et al., 2021; Seifert et al., 2017). Therefore, this study aimed to compare the CI and DL approaches from the cognitive‐perceptual perspectives and mental representation. According to the theoretical and experimental background, our hypotheses are as follows:

Hypothesis 1 According to the theoretical background of DL, (Schöllhorn, 2016) we hypothesize that the DL method would result in a higher accuracy during retention and transfer tests compared with the CI approach.

Hypothesis 2 According to the theoretical background of the perceptual‐cognitive perspective (Schack, 2004), we hypothesize that DL groups would have a more structured mental representation than the CI groups.

## 2 MATERIALS AND METHODS

### 2.1 Participants

Novice right‐handed female golfers (40 university students; mean age 21.8 ± 1.5 years) were asked to participate in this study. They were randomly divided into four groups (*N* = 10). All methods used in this study were approved by the Ethics Committee of Ferdowsi University of Mashhad. In addition, participants signed written informed consent forms before participating in this study.

### 2.2 Tools and task

A golf putting movement had to be executed on the artificial green grass (size: 4 × 7 m), using a standard golf putter and standard golf balls. The hole was marked on the grass by circles of 10.8 cm in diameter like the size of a regular golf hole. Participants putted from three different distances of 1.22, 2.44, and 3.66 m.

SDA‐M software was used to assess the mental representation structures of the participants. The SDA‐M determines the association between basic action concepts (i.e., BACs) and classifies them into clusters (Frank et al., 2016). The SDA‐M involves four steps (Schack, 2012). First, a special split procedure involving a multiple sorting task delivers a distance scaling between the BACs. Second, a hierarchical cluster analysis is used to outline the structure of the given set of BACs. Third, a factor analysis reveals the dimensions in this structured set of BACs. Fourth, the cluster solutions are tested for invariance within and between groups. BACs are representational units in long‐term memory and linked to the functional and biomechanical demands of motor actions (Schack, 2004). In this study, 16 BACs for the golf putting task were used, as described in previous studies (Frank et al., 2013, 2016). To select the BACs, the following steps were taken (Frank et al., 2013): First, movement phases were described in detail with the help of standard textbooks and the biomechanical analysis of the golf putt. The parts of the movement considered most relevant resulted in a preliminary set of 27 meaningful body postures. The 27 body postures were further rated and verified by golf experts. In the last step, a final set of 16 BACs were selected based on the experts' ratings. They are as follows: shoulders parallel to the target line (BAC1), align clubface square to the target line (BAC2), grip check (BAC3), look to the hole (BAC4), rotate shoulders away from the ball (BAC5), keep arms–shoulder triangle (BAC6), smooth transition (BAC7), rotate shoulders toward the ball (BAC8), accelerate club (BAC9), impact with the ball (BAC10), clubface square to the target line at impact (BAC11), follow‐through (BAC12), rotate shoulders through the ball (BAC13), decelerate club (BAC14), direct club head to planned position (BAC15), and look at the outcome (BAC16). Each BAC concerning one particular movement phase of the golf putt according to functional and biomechanical perspective (Göhner, 1992, 1999): preparation (BAC 1–4), backswing (BAC 5–7), forward swing (BAC 8–9), impact (10–13), and attenuation (BAC 14–16).

To assess the mental representation structures, the participants completed a splitting task. For this purpose, they were seated in front of a screen and one BAC was randomly presented at the top of the screen as the anchor concept. Then, the participants should compare the remaining BACs to the anchor concept and decide if these concepts are related during action execution.

## 3 PROCEDURE

### 3.1 Pretests

To become familiar with the task, all participants watched a video of a skilled golfer performing the putting task. Next, the experimenter introduced the participant to the splitting task. First, each participant observed a randomized list of the 16 BACs of the putt. The experimenter verbally explained the meaning of each of the 16 BACs. The splitting task was performed in front of a monitor showing the BACs of the golf putt. During the splitting task, one of the BACs was permanently displayed on top of the computer screen as an anchor (i.e., the reference concept), and 15 other BACs were displayed in a randomized order. Specifically, participants were instructed to decide whether the anchor BAC is related to another or not during movement execution. Once all BACs have been assessed about the anchor BAC, another BAC randomly took over the anchor position and the splitting task was repeated. The splitting task was completed when each BAC had been in the anchor position (Schack, 2012). Therefore, in this way, participants were required to make a total of 240 decisions (16 anchors × 15 comparisons).

As a performance pretest, the participants completed 12 putts at a target with a distance of 2.44 m from the starting point. Before the physical pretest, all participants performed three trials to warm up.

### 3.2 Retention tests

A total of 72 h after the last acquisition day, participants performed a splitting task again to determine the final level of mental representation. The performance retention‐tests were performed under two different conditions with constant (retention‐FT = performance retention‐test with fixed target) and mixed distances (retention‐VT = performance retention‐test with variable targets). In the performance retention‐test with a constant distance, participants completed 12 putting trials with 2.44 m distance from the start (equal to the pre‐test). In the performance retention‐test with variable targets, participants had to complete 12 trials with different distances of 1.22, 2.44, and 3.66 m, with the restriction that no target should be repeated twice in a row, and each target should be repeated four times.

In this phase, each participant had a short rest of 5 min between the two tests. Moreover, before the performance tests, participants executed three putts to warm up. Furthermore, the order of performing the tests was counterbalanced.

### 3.3 Transfer test

After the retention‐tests, a performance transfer test was accomplished. Participants completed 12 trials from 4 m outside the distances trained.

### 3.4 Intervention

The acquisition phase started a day after the pretest and lasted for three consecutive days. Participants of the experimental groups (except the control group) performed the putting tasks—10 blocks of 12 putts every day— for a total of 120 trials per day and 360 trials during the acquisition phase. All participants had a short break of 2 min between blocks.

A) Blocked contextual interference (BCI): For designing BCI conditions, participants practiced only one distance condition each day. Half of the group began with the shortest distance (1.22 m) on the first day, medium distance (2.44 m) on the second, and longest distance (3.66 m) on the third day, the other half of the group began with the longest distance (3.66 m) on the first day with decreasing distances on the subsequent days.
B) Random contextual interference (RCI): The random group putted randomly from three different distances (1.22, 2.44, and 3.66 m) each day, with the constraint that no distance should be repeated twice in a row, and each target should be repeated four times per block.
C) DL: The DL group was given 120 types of noise‐like and non‐repetitive movement patterns. In this group, according to the recommendations of the DL model (Schöllhorn, 1999), participants experienced variations primarily in parameters that are associated with the geometry of the movement, namely in the major joints and sensory organs of the body in each day. The changes were applied in the gradual form of DL (Henz et al., 2018) from the feet to the head or vice versa (in a counterbalanced manner between participants). That is, the noise‐like changes between two consecutive movements were systematic and mostly predictable for the participant. Examples of DL instruction could be seen in Table 1. All trials were performed from a 2.44‐m distance.
D) Control: The control group did not practice during this time.

No participant received any augmented feedback but all could see the landing location of the golf ball (extrinsic visual feedback).

**TABLE 1:** Examples of DL instructions during the practice phase.

Instruction
Stay with your feet parallel and shoulder wide, then hit the ball
Stay your feet parallel and wide apart, then hit the ball
Turn both toes extremely outward, then hit the ball
Turn the left toe inwards and the right toe outward, then hit the ball
Stay with both feet on the outer edge, then hit the ball
Stay with your right foot on the ball and with your left foot on the heel, then hit the ball
Both knees keep extended during the hit
Fix both elbow joints during the hit
Close your right eye during the hit

## 4 DATA ANALYSIS

### 4.1 Performance data

The putting performances were measured by means of the accuracy that was determined by the radial distance of the ball at the end of the putt toward the center of the whole. The data for the pretest and the retention‐test with the fixed target were analyzed using a four group (BCI, RCI, DL, and control) × 2 test (pre and retention) mixed ANOVA with a repeated measure on the last factor. In addition, one‐way ANOVA was used for the retention‐test with variable putting distances and the transfer test. For all analyses, alpha was set at 0.05. In the case of performing the post‐hoc test, the Bonferroni test was also applied. The partial eta squared was reported as effect size. Cohen (1988) has provided benchmarks to define small ($\eta_{p}^{2}$ = 0.01), medium ($\eta_{p}^{2}$ = 0.06), and large ($\eta_{p}^{2}$ = 0.14) effects (Cohen, 1988). In addition, in case of pair comparison between groups the Cohen's *d*_s_ was calculated as the effect size. Similar to Cohen (1988), the critical values for this effect size are considered as follows: small (*d* = 0.2), medium (*d* = 0.5), and large (*d* = 0.8).

### 4.2 Mental representation

After the splitting task, to specify any possible structures of BACs a hierarchical cluster analysis was used. Furthermore, a factor analysis was used to extract the dimensions of the structures. Finally, an invariance analysis compared the clusters between and within groups (Schack, 2012). For all cluster analyses conducted, an alpha level of *α* = 0.05 was chosen, which resulted in a critical value *d*_crit_ = 3.41 (Frank et al., 2013). The BACs linked above this critical value were treated as being not related, while BACs linked below this threshold were considered as a cluster. To compare cluster solutions, invariance analyses were conducted. Two cluster solutions were considered invariant when *λ* value was bigger than 0.68, while for the *λ* value lower than 0.68, two solutions were considered variant (Frank et al., 2013).

To examine the similarity of the mental representation of the experimental groups and that of expert performers, the Adjusted Rank Index (ARI) was applied (Santos et al., 2009). This index serves as an index of similarity in a range of 1, and −1. On this scale, the value (−1) indicates that two cluster solutions are different, and the value (1) indicates that two cluster solutions are the same. Indices between these extremes rank similarity between two cluster solutions. The mental representation of the expert performers consisted of five clear phases of golf putt similar to the standard phases: preparation, backswing, forward swing, impact, and attenuation.

## 5 RESULTS

### 5.1 Performance

**FIGURE 1:**
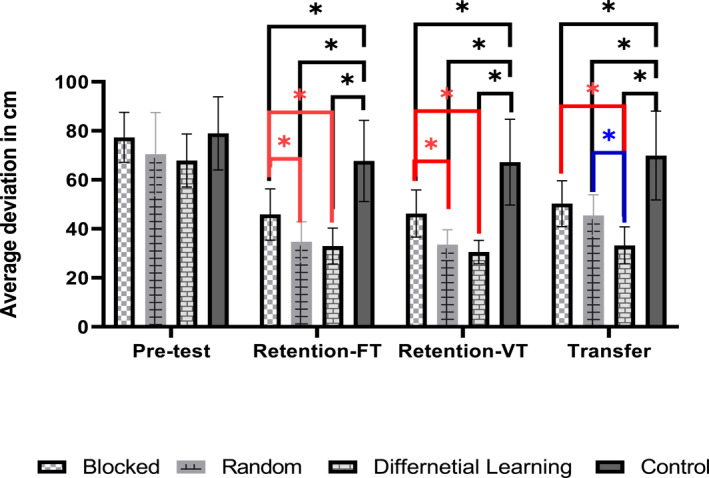
Participants' putting performance during different phases (retention‐FT = retention‐test with fixed targets, retention‐VT = retention‐test with variable targets). The vertical axis shows the deviation from the middle of the target (in cm) and the horizontal axis displays the time. The error bars show the standard deviations (SD). *: statistically significant.

The results of ANOVA for the pretest and the retention‐test with fixed target showed significant main effects of group, (*F* (3, 36) = 13.03, *p* < 0.05, $\eta_{p}^{2}$ = 0.52), and test, (*F* (1, 36) = 112.76, *p* < 0.05, $\eta_{p}^{2}$ = 0.76). Moreover, the interaction of group × test was statistically significant, (*F* (3, 36) = 4.70, *p* < 0.05, $\eta_{p}^{2}$ = 0.28). The results of the post‐hoc test for interaction revealed no significant difference between groups during the pretest, all *p* > 0.05. However, the results showed statistically significant differences between experimental groups and the control group during the retention test (see Figure 1: Retention‐FT), all *p* < 0.05 (effect sizes, Cohen's *d*_s_: BCI = 1.57, RCI = 2.38, and DL = 2.71). In addition, the RCI group and the DL group performed significantly different from the BCI group, all *p* < 0.05 (effect sizes, Cohen's *d*_s_: RCI = 1.18, and DL = 1.43). The difference between the RCI group and the DL group was not significant, *p* > 0.05. Means comparison indicated higher accuracy of the RCI and the DL groups than the other groups. Besides, the BCI group performed more accurately than the control group (means, RCI = 34.72 cm, BCI = 45.85 cm, DL = 32.94 cm, and control = 67.71 cm).

The results for the retention‐test with variable distances revealed a significant main effect of group, (*F* (3, 39) = 24.31, *p* < 0.05, *η* ^2^ = 0.67). The Bonferroni post‐hoc test for the main effect of the group indicated significant differences between experimental groups and the control group, all *p* < 0.05, (effect sizes, Cohen's *d*_s_: BCI = 1.49, RCI = 2.57, and DL = 2.86). Moreover, the RCI group and the DL group performed significantly better than the BCI group, all *p* < 0.05, (effect sizes, Cohen's *d*_s_: RCI = 1.57, and DL = 2.06). However, the difference between the RCI group and the DL group was not significant (*p* > 0.05, Cohen's *d*_s_ = 0.55). Similar to the retention‐test, with fixed distance test the results showed that the RCI group and the DL group performed more accurate than other groups, and the BCI group was more accurate than the control group (means, RCI = 33.56 cm, BCI = 46.23 cm, DL = 30.51 cm, and control = 67.21 cm).

The results of the ANOVA for the transfer test showed a significant main effect of group, *F* (3, 39) = 17.11, *p* < 0.05, and *η* ^2^ = 0.59. The Bonferroni corrected post‐hoc test for the main effect of group indicated statistically significant differences between all experimental groups with the control group, all *p* < 0.05, (effect sizes, Cohen's *d*_s_: BCI = 1.36, RCI = 1.69, and DL = 2.64). In addition, the DL group performed significantly different from the RCI and BCI groups, all *p* < 0.05 (effect sizes, Cohen's *d*_s_: BCI = 2.005, and RCI = 1.53). However, the difference between the RCI group and the BCI group was not significant, *p* > 0.05, Cohen's *d*_s_ = 0.53. Means comparison indicated higher accuracy of the DL group than other groups (means, RCI = 45.48 cm, BCI = 50.27 cm, DL = 33.23 cm, and control = 69.89 cm).

### 5.2 Structure of mental representation

**FIGURE 2:**
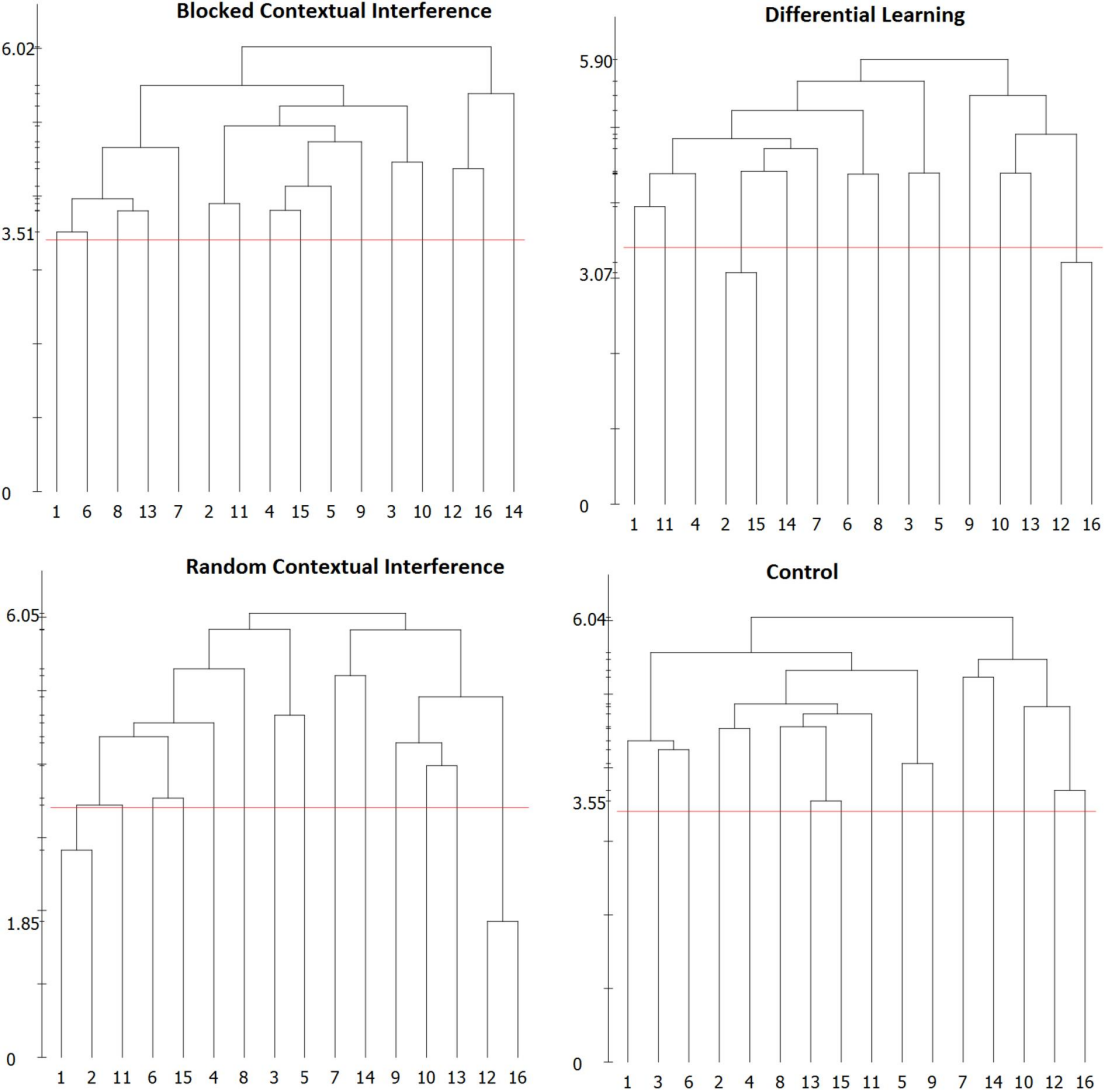
Mean group dendrograms of groups at the pretest.

In Figure 2 the cluster analysis shows little to no clustering in the mean group dendrograms of each group during the pretest. The control and blocked groups' mental representation structure revealed no significant clusters of BACs; while the RCI and DL group's dendrogram displayed two clusters at pretest, but these clusters were not related to any of the phases of movement, (BAC2, BAC15) and (BAC12, BAC16). In addition, the RCI group showed two clusters that are (BAC2, BAC11) and (BAC12, BAC16).

**FIGURE 3:**
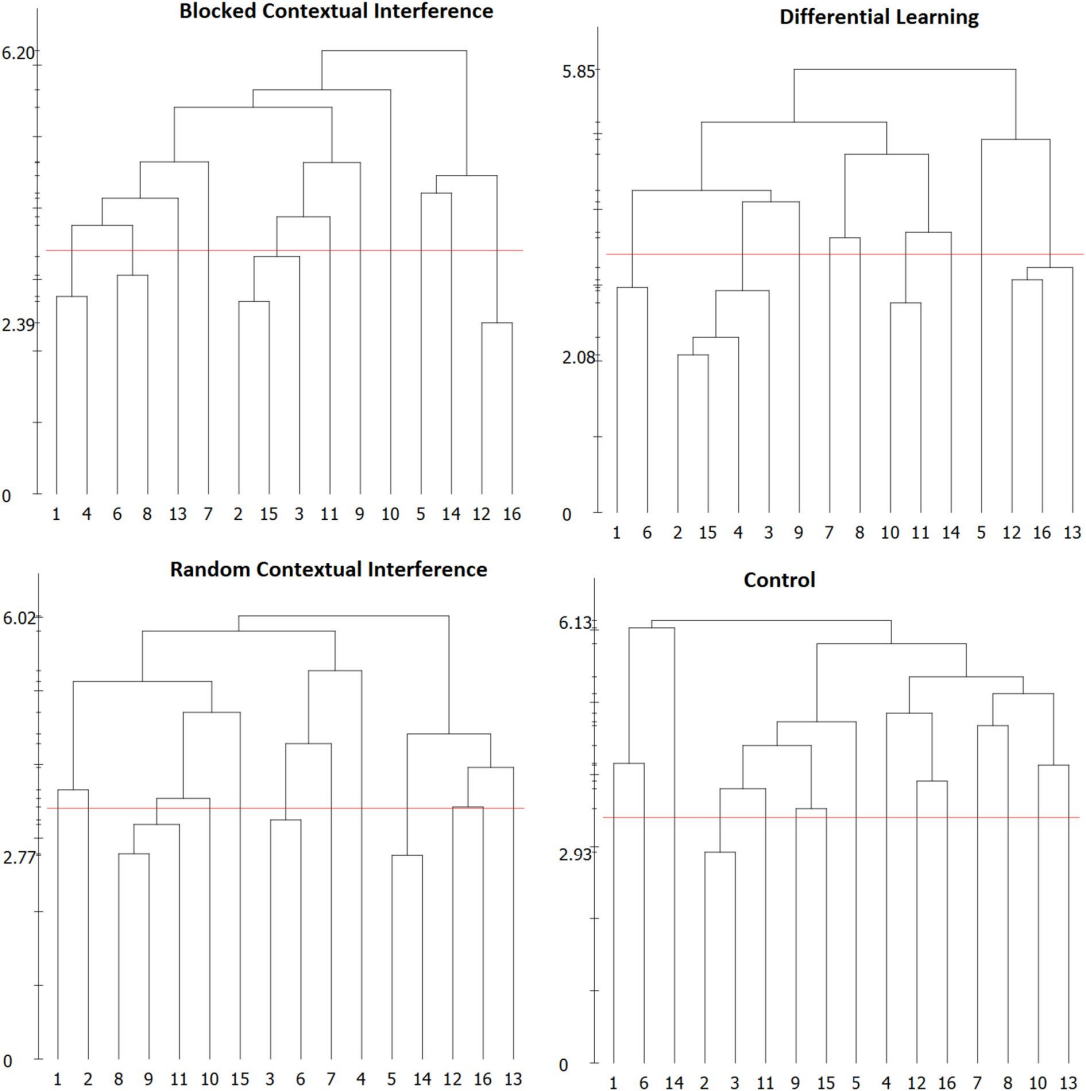
Mean group dendrograms of groups at the retention‐test.

Figure 3 displays the mean group's dendrogram at retention‐test. Significant changes were observed after intervention in all experimental groups. However, the mental representation of the control group also showed a significant cluster, (BAC1, BAC3) denoting the preparation aspect of the movement. For the BCI group, the BACs were divided into four clusters. The first cluster denoted the preparation with (BAC1, BAC4), the second cluster consisted of (BAC6, BAC8), the third cluster was related to aspects of the preparation with (BAC2, BAC15, and BAC3), and the last cluster was a non‐functional unit with (BAC12, BAC1). For the RCI group, three clusters were observed (BAC8, BAC9, BAC11), (BAC3, BAC6), and (BAC5, BAC14). The first cluster was related to the aspect of the forward swing; the two other clusters were not related to the movement phase during the putt. Thus, this relation is based on superficial rather than on functional characteristics. Lastly, for the DL group four clusters were observed (BAC1, BAC6), (BAC2, BAC15, BAC4, BAC3), (BAC10, BAC11), and (BAC12, BAC16, BAC13). In detail, the first cluster was a non‐functional unit, the second cluster represented the preparation phase of the movement, and the last two were related to the impact phase during movement execution.

The analyses of invariance were utilized to receive information about differences between cluster solutions, from pretest to retention‐test (Schack, 2012). Analysis of invariance showed significant differences between pretest and retention‐test for all groups (all *λ* < 0.68). This can be interpreted in a way, that all groups showed changes in the structure of mental representation.

The mental representation structures of the groups were compared with the mental representation structure of the skilled group. In particular, the adjusted rand index (ARI) showed that the experimental groups became more similar to the mental representation structure of skilled golfers as a result of practice. The ARI from pretest to retention‐test were as follows: blocked (from 0 [zero] to 0.13), random (from 0.13 to 0.15), and DL (from 0.05 to 0.32). Furthermore, in comparison to the reference structure, the control group's structure showed only a slight trend toward the skilled group over time (from 0 to 0.06).

In general, the results showed that in the retention‐test, the DL group had the most similarity to the reference structure.

## 6 DISCUSSION

The aim of this study was to compare the effects of a 3‐day CI and DL intervention on performance and mental representations in a golf putting task. The experimental design with a pretest followed by three consecutive days of intervention and a 3‐day break before the retention test followed, does not allow to interpret the data with respect to the first part of the CI effect that relates to a posttest. The retention‐test results of the putting performance showed learning progress in all groups. In accordance with the second part of the CI effect that relates to the retention phase, the BCI group (fixed and variable targets) performed worse than the RCI group. The results revealed more learning progress after random when compared to blocked CI practice. Although previous studies, primarily conducted on movements with sDGF, argued that variations within the same GMP do not create sufficient interference to enhance retention or transfer performance (Sekiy et al., 1996; Sekiya et al., 1994; Wulf et al., 1993), our findings are in line with (Fazeli et al., 2017; Shea et al., 1979), who showed that changes in the parameters of a class of action that require the same GMP could create the advantageous learning outcome. Whether this is related to the number of degrees of freedom of the movements (Apidogo et al., 2022) or related to other modulating parameters, for example, different time pressure (Wright et al., 1991), it needs further research.

One important finding of this study is that the DL group performed better than the RCI, BCI, and control groups on all tests that followed the pretest. However, the difference reached statistical significance only on the transfer test. The extent to which this can be attributed to the relative short intervention duration of 3 days requires further research. Comparable studies on sports movements with 6 weeks (Schöllhorn et al., 2006) or 3 months (Oftadeh et al., 2022) of intervention indicate a magnification of the positive effects with increasing duration of the intervention. For even more differentiated conclusions, it would be necessary not only to look for the movement outcome but also to compare the variations of the movements biomechanically during the randomized CI sequence with the variations during the DL intervention in terms of volume and structure of the noise as it has been suggested in previous studies (Burdack et al., 2020; Horst et al., 2020). A comparison of the present study with the study of Schmidt (Schmidt et al., 2021) is problematic, as with the latter, increasing random CI was applied, whereas here constant random CI from the beginning was used. Both studies in the DL group did not amplify observed fluctuations in each athlete as demanded by the stochastic resonance principle but applied the same variation to everyone (Schöllhorn et al., 2009). Nonetheless, the CI and the DL approaches produced increased noise in motion executions. One provokes increased motion noise by varying putting distances, while the other achieves higher levels of noise by adding stochastic perturbations to each movement (Schöllhorn, 2016). To further explore the commonalities and differences of both approaches, a detailed biomechanical analysis of the movements during training would be necessary. Whether the increase of noise becomes detrimental by passing a threshold or whether a plateau is reached, must be shown by future research. Interestingly, the BCI and RCI performances in the transfer test did not differ statistically significantly. This could be assigned to the characteristics of the transfer test. The transfer test was executed in a distance that was outside the distances during training, which, in terms of neural nets corresponds to extrapolation in difference to interpolation (Schöllhorn, 1999). Differences in transfer tests can also be observed in earlier studies, depending on whether the transfer test took place inside or outside the trained area. In majority, no differences were found between BCI and RCI in a transfer test that was outside the trained range, (Memmert, 2006; Vera et al., 2008) whereas when the transfer test was within the trained range, RCI showed superior results in comparison to BCI (Vera et al., 2003). From this point of view, the better transfer performances of the DL group are somehow extraordinary, as they had only practiced at 2.44‐m distance. Together with the observation that the DL group outperformed BCI and RCI in the transfer test, both phenomena provide additional arguments in favor of the DL model, as the amplification of the fluctuations led to the coverage of a larger solution space and increased the probability of taking advantage of interpolation instead of extrapolation. From an information theory perspective, the added noise in the DL group provided supplemental information that supports self‐organization of the neuro‐muscular system. Stochastic perturbations described as forms of noise, for example, quantified by the frequency spectrum of entropy, were also suggested to unite different approaches for motor learning and their practical consequences (Römer et al., 2009; Schöllhorn, 2005; Schöllhorn et al., 2006). Therefore, different approaches to motor learning such as repetitive learning, CI, variability of practice, and DL can be considered as having different amounts or colors of noise based on the amount of variation during movement acquisition, from low stochastic perturbations (repetitive learning) to high stochastic perturbations (DL) (Beckmann et al., 2010; Römer et al., 2009). In this regard, DL theory predicts that the correlation between the amount of noise and performance is similar to a U‐inverted curve. When the resonance curve was suggested first in 2005 (Schöllhorn, 2005), the maximum amount of learning rate was assumed to be close to the noise associated with DL in its narrow sense (Römer et al., 2009) because at this time, the CI and variability of practice approach just turned out not to apply to movements with many degrees of freedom (Brady, 2004; Wulf et al., 2002). In this way, the noise generated by the movement during learning increases signal recognition and the learning rate (Schöllhorn, 2016). Such stochastic perturbations enable the individual to perform optimally in new situations (Beckmann et al., 2010). Accordingly, it is possible that due to the nonoptimal amount of noise around a single specific movement that is triggered by switching between different distances during random practice schedules, the CI group did not perform as good as the DL group in the transfer test.

On the other hand, from an artificial net point of view, the bigger variations in DL seem to prepare the learners' neural nets for a wider spectrum of tasks than the three distances in random order applied in CI learning. Nevertheless, the random sequence of distances seems to lead to positive effects in comparison to a blocked schedule. Whether these effects are due to the constant changes between distances and can be associated with cognitive explanations or whether they can be traced back to the increased movement variability for each putting movement in each distance that is accompanied when switching between the three distances (Janssen et al., 2010) needs further research.

The results on mental representation are in line with previous studies that revealed the random CI group had a more structured mental representation than the blocked CI group (Fazeli et al., 2017). However, in the present study, this difference was not very distinct. Several reasons are possible for the lack of a clear difference between the two groups. First, it should be noted as a limitation that the random CI group already had a significantly higher score in mental representation structure during the pretest, which may be a possible reason for lacking the differences between the RCI group and the BCI group. Typically, it is assumed that with increasing the learning level the progresses decrease. Another possible reason could be the duration of the acquisition phase. While in the study by (Fazeli et al., 2017), participants practiced for 1 week with more sleeping units (7 days, 180 trials each day), in this study, the acquisition however lasted only three consecutive days (120 trials each day). According to (Boutin et al., 2010), the amount of practice is important to create the CI effect. Thus, it was possible that due to the short duration of acquisition and consequently small number of trials, the mental representation of the RCI group did not differ significantly from the BCI group.

This study also showed that the DL led to a more structured mental representation after intervention than the other practicing schedules (random CI and blocked CI). This finding is in line with the cognitive action architecture approach (Schack, 2004; Shea et al., 1979). According to this view, actions are designed, executed, and stored in memory as representations of predicted perceptual effects. Several studies have shown that skilled people, in addition to higher performance, also differ in their mental representation structure from beginners (Schack et al., 2006; Weigelt et al., 2011). It has also been pointed out that the structure of mental representation is influenced by the motor learning approach (Fazeli et al., 2017; Frank et al., 2016). The answer to the question of why the strongest mental representation is formed in the DL group may have to do with the nature of the practice. Previous research on DL has noted that in terms of memory function, this type of practice involves more WM areas in the kinesthetic and tactile sensory area that are related to the processing of somato‐sensory information and less cognitive information (anterior areas) (Henz et al., 2018). Kinesthetic, proprioceptive, and tactile WM may have a positive correlation with the differentiation of mental representation in long‐term memory (Kim et al., 2019). Activating areas related to WM could enhance the formation of mental representation of action in long‐term memory. In this context, it would be interesting to see in the future whether the mental practice using DL leads to similar mental representations or whether the movements are needed to provide the dominating mechanical and accompanying visual stimuli. Furthermore, it would be of interest to see to what extent individual differences in a forensic sense arise within the given groups according to the criteria of uniqueness and persistence (Horst et al., 2020). However, studies with cross‐over and single case designs would be a prerequisite for this (Schöllhorn, 1993).

### 6.1 Strengths and limitations

In general, according to the results of this investigation, DL provides evidence to be more effective in generalization and transfer of a motor task to new conditions than CI. The limitations of the study are given, epistemologically, by the boundary conditions of the study design itself. Due to the design (including, the limited number of participants, data acquisition‐lack of recording the acquisition data, testing, intervention, data processing, etc.) no claims for generalization are allowed. For further development and corroboration of the models on variable learning biomechanical analysis in combination with investigating the neurophysiological responses are recommended. At least, according to Fishers's interpretation of statistically significant results, the findings encourage to continue investigating DL.

## 7 CONCLUSIONS AND PRACTICAL APPLICATIONS

Given the novelty of the approach of comparing learning approaches on the basis of internal representations, more questions arise than could be answered. In line with the theory of DL, one question could focus on individual differences in reactions depending on the learner's learning history. The question about the situation‐dependency of the learning process points in a similar direction. What influence do emotions or fatigue have here? Generally, the results of this study showed again that DL could result in higher accuracy in transfer situations than in CI. In addition, results showed that DL could provide a more structured mental representation compared with CI. Recommendations for practice based on a single study should not be made for reasons of scientific theory. However, as this is a further study that demonstrates the advantages of DL, it can be recommended that coaches should experiment with more variable training options and no longer regard deviations from predetermined ideals as errors that can be ruled out. According to the challenge point framework (Guadagnoli et al., 2004) and the U‐inverted shape of correlation between noise and progress in motor learning provided by the DL approach (Schöllhorn, 2005), the amount of noise should be proportional to the learner's skill level. Accordingly, recommending using DL in its most extreme version with highest level of noise to teach novices with already high level of internal noise, would not be in line with these theories. For beginners or children, repetition often has enough noise for successful learning, but only at the beginning.

## CONFLICT OF INTEREST STATEMENT

There is no conflict of interest to declare.

## Supporting information

Supporting Information S1
